# The Experience of Failed Humor: Implications for Interpersonal Affect Regulation

**DOI:** 10.1007/s10869-014-9370-9

**Published:** 2014-06-26

**Authors:** Michele Williams, Kyle J. Emich

**Affiliations:** 1 ILR School, Cornell University, Ithaca, NY 14853 USA; 2 Graduate School of Business, Fordam University, New York, NY USA

**Keywords:** Interpersonal affect regulation, Humor, Affect-related individual differences, Affective perspective taking, Gender differences, Efficacy, Motivation to persist, Narrative methodology

## Abstract

**Purpose:**

The purpose of this study was to investigate failed interpersonal affect regulation through the lens of humor. We investigated individual differences that influenced people’s affective and cognitive responses to failed humor and their willingness to persist in the interpersonal regulation of positive affect after a failed attempt.

**Design/Methodology/Approach:**

Using well-established autobiographical narrative methods and surveys, we collected data at two time points. All participants (*n* = 127) received identical surveys at time 1. At time 2, they were randomly assigned to complete a narrative about either successful or failed humor as well as a second survey.

**Findings:**

Using moderated regression analyses and SEM, we found significant differences between our failed and successful humor conditions. Specifically, individual differences, including gender, affective perspective taking, and humor self-efficacy, were associated with negative reactions to failed humor and the willingness of individuals to persist in the interpersonal regulation of positive affect. Moreover, affective perspective taking moderated the effect of gender in both the failed and successful humor conditions.

**Implications:**

Our results suggest that failed humor is no laughing matter. Understanding individuals’ willingness to continue in attempts to regulate the affect of others contributes to the comprehension of an understudied phenomenon that has implications for interpersonal behavior in organizations such as helping, group decision making, and intragroup conflict.

**Originality/Value:**

Studies of interpersonal affect regulation often focus on people’s ability to successfully regulate others’ emotions. In contrast, this is the first quantitative study to explore factors that influence individual’s willingness to persist in interpersonal affect regulation after failure, and to investigate how individual differences influence the personal outcomes associated with failed attempts.

## Introduction

Active efforts to regulate the feelings of others are a ubiquitous part of social life. People deliberately try to influence the emotions of others for a variety of purposes ranging from self-interested material gain to altruistic social support. For instance, subordinates may use humor to make their superiors like them and feel happy (Cooper 2005), competitive negotiators may try to induce guilt or empathic concern to make their counterparts give up resources (Thompson 2011), abusive leaders may induce fear to gain compliance from subordinates (Harvey et al. 2007), and friends may reframe painful events in ways that reduce stress and bring relief to each other (Niven et al. 2009).

Despite the growing interest in the domain of interpersonal affect regulation (e.g., Niven et al. 2012b), the field lacked a common framework until recently (see Niven et al. 2009 for review). In order to build this framework, previous research has focused on establishing that individuals can successfully influence the emotions of others and on the personal and interpersonal consequences of successfully regulating others’ affect when required to do so (Niven et al. 2009; 2012a, b; Grandey et al. 2005). For example, on the negative side mandated interpersonal affect regulation (i.e., emotional labor) can cause job strain and burnout (Grandey et al. 2005). However, more naturally occurring interpersonal regulation of positive affect has been associated with better client relationships and personal affective well-being over time as well as trust and stronger relationships among coworkers—outcomes that, in turn, may reduce conflict and improve workgroup cohesion (Niven et al. 2012a, b). Despite the scholarly and practical relevance of interpersonal affect regulation, scholars have paid scant attention to the consequences of *failed* interpersonal affect regulation (but see Francis et al. 1999). Despite this lack of research attention, interpersonal affect regulation is a skilled behavior and nearly everyone experiences failed attempts at regulating others’ feelings (Francis et al. 1999).

In this study, we inductively examine the experience of failed interpersonal affect regulation through the lens of humor. We use the lens of humor to develop theory about how people experience and respond to failed interpersonal affect regulation and what individual-level factors influence their response. Whereas interpersonal affect regulation refers to the process of consciously trying to change the feelings of a target individual (Gross and Thompson 2007), we define failed interpersonal affect regulation as the process of consciously, but unsuccessfully trying to change the feelings of a target individual such that the target’s emotional expression either remains unchanged, changes less than expected (e.g., humor that receives a chuckle versus a belly laugh), or changes in a direction other than that intended (e.g., trying to improve someone’s mood but receiving a reaction that indicates anger or anxiety).

Scholars have argued that intentional humor involves all of the core elements of interpersonal affect regulation (Francis 1994; Francis et al. 1999; Niven et al. 2009). In turn, we argue that humor provides a relevant and useful lens for examining failed interpersonal affect regulation for four reasons. First, humor is intended to influence positive affect. Humor has been defined as remarks or non-verbal behaviors that are intended to elicit the feeling of amusement and are perceived by the targets as an intentional act (Robinson and Smith-Lovin 2001; Cooper 2005).^1^ Further, humor regulates affect through two well-established pathways. It redirects the target’s attention toward a potentially amusing action or communication and influences the target’s body including the facial expressions, bodily postures, and motor movements associated with joy (Koole 2009).

Second, humor does more than influence feelings by creating amusement in the self and others. It also generates “positive emotions among members of an interacting group by bonding them and/or reducing an external threat” (Francis et al. 1999, p. 171). For example, humor has been shown to decrease the social distance between people (Masten 1986; Sherman 1988) as well as increase bonding and rapport (Romero and Cruthirds 2006). Thus, humor plays an important role in the creation and maintenance of interpersonal relationships.

Third, in contrast to other forms of interpersonal affect regulation, which may or may not provide discernible feedback, people receive immediate and undeniable feedback about the success or failure of their humor attempt—i.e., targets either laugh or they do not. For example, one medical provider stated, “I tried to make a joke to ease the situation, and it just fell flat…[I] could tell from their body language that they didn’t think it was funny” (Francis et al. 1999, p. 170).

Fourth, extant research provides evidence that humor can act to regulate individual emotion (Mesmer-Magnus et al. 2012). For example, individuals use humor to reduce the impact of negative emotions (Samson and Gross 2012). Moreover, humor can alleviate boredom and frustration (Duncan 1982; Pryor et al. 2010) and be used as a coping mechanism to attenuate the influence of stress encountered in interpersonal situations (Martin et al. 2003).

In our view, the common dominator in all experiences of failed interpersonal affect regulation is the negative affect experienced by the agent. At the most basic level, failed interpersonal affect regulation reflects goal interruption, which generates negative affect (Mandler 1975). However, such failed regulation, especially in the case of humor, can also represent a threat to the agent’s social bonds with the target (Francis et al. 1999; Romero and Cruthirds 2006). Thus, in contrast to the goal of a humor attempt, which is typically to increase the level of positive affect in an interpersonal interaction, failed humor may lower positive affect and generate negative affect in the agent.

Given the importance of positive affect for interpersonal interactions such as helping and maintaining social bonds (Fredrickson 2001; Niven et al. 2012a), for task-related skills such as decision making and creativity (Estrada et al. 1997; Fredrickson and Branigan 2005; Isen 2008; Isen et al. 1987), and for individual well-being (Niven et al. 2012b), we seek to better understand the effects of failed interpersonal affect regulation by explicitly examining people’s reactions to failed humor attempts.

Our paper is organized as follows. First, we describe the focus of our inductive inquiry. Then, we present our methods and analyses. We then adapt the results of the analyses to inductively establish a general theoretical model of factors influencing an agent’s response to failed interpersonal affect regulation and conclude with a discussion of the theoretical and practical implications of our model for interpersonal affect regulation in organizations.

## Focus of Inductive Inquiry

Our main goal was to examine how individual differences may buffer agents from the negative experiences associated with failed humor (e.g., increased negative emotion and decreased self-efficacy) and bolster their self-reported willingness to make new attempts at increasing the positive affect of others. In order to do this, we first investigate baseline information about failed humor. We uncover and document, from the perspective of the agent, the range of affective, cognitive, and behavioral indicators that are associated with failed humor.

In particular, we focus on guilt, laughter, new humor attempts, and humor self-efficacy. Whereas laughter is an intended outcome of humor (Koole 2009), guilt is a likely outcome of failed humor. Guilt may occur because failed humor can offend targets (Francis et al. 1999), and guilt is an emotion associated with the desire to redress wrongs (Baumesiter et al. 1994; Bohns and Flynn 2012). Moreover, new humor attempts are important because they maintain social interactions and allow for the possibility of increased positive affect, which has benefits for individual well-being, helping, and maintaining social bonds (Niven et al. 2012a, b).

Finally, a great deal of evidence suggests that agent’s confidence in their own ability (i.e., their humor self-efficacy) may be impacted by the experience of failure (Bandura 1997). Domain-specific forms of self-efficacy have been shown to increase motivation toward goals within a specific domain (Bandura 1997; Bandura and Locke 2003), and thus, humor self-efficacy may influence people’s willingness to persist in interpersonal affect regulation. Moreover, our focus on humor self-efficacy is unique because the potential importance of efficacy in the domain of interpersonal affect regulation has not received research attention (Mayer et al. 2008).

Next, we looked for factors that buffer agents from the negative effects of failed interpersonal affect regulation and motivate them to continue in their effort to regulate the affect of others. Specifically, we focused on guilt propensity and perspective taking because both of these variables are associated with relationship repair (Batson et al. 1995; Baumeister et al. 1994; Bohns and Flynn 2012; Davis 1996) and responding to failed humor may require repairing the interaction and/or relationship (Francis 1994; Francis et al. 1999). For instance, affective perspective taking, i.e., imagining other peoples’ feelings from their point of view, is an empathy-related process (Davis 1996) that may increase agents’ desire to help others feel better (Batson et al. 1995) and thus may also increase their willingness to persist in interpersonal affect regulation after experiencing failure. Similarly, guilt, “an individual’s unpleasant emotional state associated with possible objections to his or her actions, inaction, circumstances, or intentions,” is associated with relationship repair behaviors (Baumeister et al. 1994, p. 245; Bohns and Flynn 2012) and thereby may increase the motivation to persist in interpersonal affect regulation after experiencing failure.

Finally, we investigated gender as a substantive control variable and possible individual difference factor for two reasons. On the one hand, studies suggest that humor is more important to men than it is to women (Bressler et al. 2006). On the other hand, women consistently outperform men on experimental tasks related to interpersonal perceptual accuracy (Hall and Schmid-Mast 2008) and are also more accurate in judging the meaning of non-verbal cues conveyed by others (Hall 1978; Hall and Schmid-Mast 2008; Hojat et al. 2002; Salovey and Mayer 1990; Woolley et al. 2010). These findings provide evidence that gender may influence the processes surrounding interpersonal affect regulation, and specifically men’s and women’s reactions to failed attempts.

In brief, our study was designed to use humor episodes to inductively examine the psychological experience of failed interpersonal affect regulation—the intrapersonal affective, cognitive, and behavioral consequences of such episodes. Given the difficulties of experimentally examining the psychological experience of *failed* humor (Francis et al. 1999), the present study elicited autobiographical narratives in which participants recounted interpersonal encounters in which they had used humor and it had either failed (failed humor condition) or succeeded (successful humor condition). Autobiographical narratives have proven useful in studying a range of phenomena that resist laboratory simulation, such as romantic losses and rejections (Baumesiter et al. 1993), hurt feelings (Leary et al. 1998), and victim and perpetrator memories (Baumesiter et al. 1990; Stillwell and Baumeister 1997). Moreover, because “humor is a specific and easily recognizable form of interaction,” it is particularly amenable to recall-based methods such as autobiographical narratives (Francis et al. 1999, p. 156). Finally, the use of autobiographical narratives allowed us to draw on a broad range of responses that are likely to inform our understanding of failed interpersonal affect regulation.

## Method

### Participants

One hundred and twenty-seven undergraduate students (74 % female) participated in the study. These participants were recruited through the online subject pool at a large northeastern research university. All participants took part in exchange for $15.

### Procedure

Participants completed questionnaires individually, in two phases. The first phase asked all participants to complete the same online questionnaire, which included individual differences on measures of trait affectivity (including guilt and affective perspective taking), humor self-efficacy, and background demographic information. In the second phase, which occurred 1 week after the first phase, participants (both men and women) were randomly assigned to one of the two conditions (i.e., failed humor narrative versus the successful humor narrative condition). During the second phase, participants wrote a randomly assigned narrative and then responded to a second questionnaire that both asked participants to recall their state affectivity and humor self-efficacy following the attempt and asked detailed questions about the events that were recounted.

#### Phase 1 Measures

In order to measure a range of responses to interpersonal affect regulation in the form of successful and failed humor attempts, a wide variety of measures were given. These measures focused on the attitudes, cognitions, and behaviors of the participants. Initially, participants received an online questionnaire regarding their humor self-efficacy, trait affectivity [positive affect (PA), negative affect (NA), trait guilt, and affective perspective taking], and demographic background. The 11-item Humor Production and Social Uses of Humor subscale of the Self Sense of Humor Scale was used to assess perceived humor self-efficacy. The scale was deemed appropriate to measure humor self-efficacy, or confidence in one’s ability to be humorous, because it explicitly asks participants to rate their confidence in their ability to use humor to accomplish a variety of ends (e.g., amuse others, entertain friends, make others laugh, ease a tense situation). Moreover, this measure has been found to be positively associated with peers’ views of humor ability in laboratory settings (Lefcourt and Martin 1986). Affective perspective taking, which focuses specifically on imagining how others are feeling from their point of view (Davis 1996), was measured with Williams’ (2011) 3-item measure of affective perspective taking. Participants were asked on a scale of 1 (*Not characteristic at all*) to 5 (*Very characteristic*), the degree to which they (1) try to understand others’ feelings, (2) think about how they would feel if in the place of others, and (3) try to imagine what emotions others are feeling. Trait affect was measured using items from the 20-item positive and negative affect schedule (PANAS, Watson et al. 1988) and the six-item guilt subscale of the PANAS-X (Watson and Clark 1994). Participants were given a list of the affect-related words and were instructed to “indicate the extent to which you generally feel this way, that is, how you feel on average.” Finally, participants answered demographic questions concerning their ethnicity and sex. Sex was recorded with a categorical variable (1 = female, 0 = male). We used two categorical variables to capture race/ethnicity. These variables reflected the two largest ethnic groups in our sample: White (1 = Caucasian, 0 = all other) and Asian (1 = Asian, 0 = all other). The baseline reference group included African-Americans and Latinos.

#### Phase 2 Measures

One week after all participants completed the phase 1 questionnaire, they were randomly assigned to the failed or successful humor condition and received the appropriate questionnaire. Random assignment was used to address sampling bias because subjects randomly chosen from a given population have an equal chance of displaying any given characteristic (Kirk 2012). It also avoided biases associated with individuals whose characteristics may have led them to select to write about failed versus successful humor. For example, individuals with lower humor self-efficacy may have chosen to write about a failed humor event.^2^

In order to gather data on participants’ self-reported thoughts and feelings related to humor attempts, participants were first asked to type an autobiographical narrative about a humor attempt. In the successful humor condition, they were asked to, “Please think of a story about a time in the past 6 months when you tried to be humorous (i.e., make someone laugh), and you got the reaction you were looking for,” whereas those in the failed humor condition were asked to, “Please think of a story about a time in the past 6 months when you tried to be humorous (i.e., make someone laugh), and you did not get the reaction you were looking for.” We included the phrase “to make someone laugh” in our instructions for clarity and then coded narrative responses for intentions to change the targets’ inner feelings as well as change their outward emotional display. Although recall biases can emerge when asking participants to remember an event from the past, these biases usually occur when participants recall some information related to a specific event, to the neglect of other applicable information. Using a narrowing approach, asking participants to recall individual components of an overall event has been found to attenuate these biases (Caruso et al. 2006). Therefore, to reduce instances of recall bias, we structured the second survey by asking participants to separately address (a) what prompted the attempt, (b) how and when they made the attempt, (c) what impact the attempt had, and (d) how the agent reacted to the target’s response. Additionally, to help ensure participants recalled an actual event, we asked them to write the initials, age, gender, and their relationship to the target of the humor attempt. However, because we understand that recall biases may still have persisted, we explicitly test for them and further address the implications of our findings in the discussion.

Participants were then asked whether they had a goal in mind when making the attempt, what transpired following the attempt, and why the attempt did, or did not, receive the intended response. After typing their stories, participants completed a detailed set of Likert scale rated survey items about their autobiographical humor narratives. The items were used to capture participants’ psychological experience of a failed/successful humor episode—their feelings, cognitions, and behavioral responses.

#### Post-Narrative Measures: Feelings

Participants filled out questionnaires measuring their state affect and self-esteem. In order to measure state affect, the PANAS and PANAS-X were again utilized (Watson et al. 1988; Watson and Clark 1994). Specifically, this time, participants were instructed to “please think back to your feelings directly following the humor attempt, indicate the extent to which you feel the following adjectives describe your feelings of yourself after the humor attempt.” Additionally, we captured self-esteem by having participants rate themselves on Leary et al.’s (1998) measure of six positive and negative self-relevant items: stupid, undesirable, unlikeable, unattractive, intelligent, wise, likeable, incompetent, attractive, competent, foolish, and desirable (1 = *Not at all;* 5 = *Extremely;* see Leary et al. 1998).

#### Post-Narrative Measures: Cognitive Responses

Participants were explicitly asked about their humor self-efficacy following the attempt using items from Lefcourt and Martin (1986). Attributions were assessed using narrative content analysis (see below).

### Narrative Content Analysis: Cognitive and Behavioral Reponses

Three undergraduate research assistants read the 127 narratives and classified each humor attempt based on four dimensions. The research assistants, who were blind to the purposes of the study at the time they coded the narratives, were trained and then asked to assess (a) the type of humor attempted, (b) the goal of the humor attempt, (c) the behavior of the agent following the attempt, (d) the reaction of the target following the attempt, and (e) the attribution the agent made for the success or failure of the attempt. These coding categories were theoretically derived as manipulation checks (type and goal of humor) or as foci of the study (behavior and cognitive attributions).

However, some of the coding dimensions for each category were developed inductively. The second author read through all of the narratives to identify emergent coding dimensions for each code category (Miles and Huberman 1994; Strauss and Corbin 1998). These emergent codes were added to codes derived from the literature. The first author then applied the coding scheme to a subset of narratives and together the authors revised the coding dimensions before training the research assistants. Although the literature on attribution theory identifies the importance of internal versus external attributions, this inductive process allowed us to uncover that participants distinguished between two different types of external attributions (those related to the target of the humor and those related to the humor method).

The research assistants, who were blind to the purposes of the study, coded all five categories for each story. First, the research assistants were asked to assess the type of humor attempted, i.e., whether the attempt made was positive or negative. Negative humor was defined as situations in which the agent attempted to be funny by putting down the targets, teasing them, or using sarcasm. For example, one participant wrote that he or she, “mocked (his or her friend), in a voice imitating her right after she said something stupid (ID# 1384).” This was coded as negative humor. Although these attempts were negative in that they included some level of provocation or criticism, they were intended to be humorous, to elicit positive affect, i.e., “to make the target laugh.” Even acts such as teasing, which can be used aggressively, also can be used to elicit amusement (Keltner et al. 2001). For example, Keltner et al. (2001, p. 234) note that when teasing contains “provocations *accompanied by* numerous off-record markers” such as hints, questions, rhetorical questions, or metaphors, it “will be perceived as playful.”

We coded for the type of humor (positive or negative) to see whether differences in the type of humor events individuals were likely to recall in the successful versus failed condition explained any of our findings about failed humor. We surmised that because teasing and other negative humor strategies can be perceived as aggressive when subtleties such as off-record markers are not used properly (Keltner et al. 2001), individuals may be more likely to fail when they use negative strategies than when they employ more positive types of humor. In our sample, 42 participants reported a negative humor attempts and 85 reported a non-negative humor attempts (*n* = 127).

Second, the research assistants were asked to judge the goal of the humor attempt, i.e., whether the attempt entailed trying to increase the target’s positive affective bonds of friendship with the agent or trying to increase the target’s general positive affect. For example, someone with a goal of increasing positive affective bonds of friendship wrote that he or she attempted to be funny because he or she “wanted to maintain our friendship since we don’t get to see each other that much (ID# 1220).” Participants who tried to change the target’s positive affect made statements such as they were “attempting to use humor to make him feel better after a stressful day” (ID# 1227), “trying to put him in a better mood” (#1337) or trying “…to make him laugh and feel better (1220).” We coded for the goal of the humor attempt to confirm our underlying assumption that humor was being used to regulate the feelings of others. In our sample, 99 participants had the goals of “increasing positive affect,” 20 had the goal of “increasing affective bonds” and eight were coded as other.

Third, all narratives were coded for the behavioral response of the agent, i.e., whether, following the attempt, the agent laughed, made a new and different attempt at humor, repeated the same attempt, apologized for the attempt or did nothing. For example, an agent making a new different attempt stated that after the attempt they “followed up by making another joke to try to get [the target] to laugh again (ID #1240).” Conversely, someone who repeated the same attempt stated, “I continued telling the same story, but tried to make it more ridiculous so she would laugh (ID #1335).” One participant that was coded as apologizing stated, “Two of the people at the table chuckled but said that the joke was mean spirited. I apologized… (ID# 1307).” Finally, one participant who was coded as doing nothing after the attempt stated that after the attempt they “just didn’t make any more comments (ID #1337).” We coded for the agent’s behavior following the attempt to uncover patterns of behaviors associated with failed humor.

Fourth, the research assistants coded each narrative for the behavioral response of the target. Target’s behavioral responses were initially coded into two categories: responded as expected versus unexpected. These categories were coded by all three research assistants. To gain additional insight into the type of expected and unexpected responses that targets made, one research assistant and one coauthor went back through the narratives. The three categories that emerged from the data included the target laughed, the target was bothered by the attempt, and the target continued the interaction. Specifically, targets were only coded as laughing if this was explicitly mentioned in the response. For example, one person recalled, “(the target) laughed and thanked me for listening (ID# 1236).” Next, humor attempts were coded as bothersome when the target engaged in a specific negative behavior aimed at the agent. For example, one person recalled that the target, “certainly did not smile. They didn’t really look at me either. They just kept their eyes on the TV. Occasionally, they might say something curt, but it wasn’t anything that recognized my attempt to be fun and humorous (ID# 1211).” Finally, whether the target made an explicit attempt to continue the interaction was cataloged. For example, one person recalled that the target “smiled and told me an anecdote as well (ID# 1303).”

Finally, the research assistants coded participants’ cognitive attributions for the outcome of the humor attempt, i.e., whether the outcome of the attempt was due to themselves (an internal attribution), the target (an external attribution), or the method of humor attempt (an external attribution). Attributing the success or failure of the attempt to the agent was characterized by participants explicitly stating that something inherent in them had led to the success or failure of the attempt. For example, one participant said, “I was quite funny (ID# 1237),” whereas another said a joke failed because, “I have a different sense of humor from other people (ID# 1247).” Both of these examples were coded as internal self-attributions. Conversely, participants who attributed the success or failure of an attempt to target(s) made comments such as, “It failed because people are too stressed (ID# 1209),” and “my friends are dumb (ID# 1249).” Finally, those attributing failure to the attempt itself explicitly stated that something inherent in the attempt, as opposed to themselves, or the target, caused the outcome. For example, one participant stated, “The joke was inappropriate (ID# 1212).” We coded for the agent’s attributions following the attempt to uncover patterns of attributions associated with failed humor. All disagreements in classification were resolved by discussion among the research assistants. Finally, Shrout and Fleiss’ (1979) intraclass correlations (2, 1) were calculated to assess interrater reliability based on the premises that each judge rated their own random sample of targets and the judge’s data was combined for analysis. Interrater reliability exceeded 0.80 for all categories.

## Results

### Descriptive Statistics and Manipulation Checks

The descriptive statistics for all of our scales are presented in Tables 1 and 2. The reliabilities of all scales were above the 0.7 criteria established by Nunnally (1978).

**Table 1:** Descriptive statistics and zero-order correlations for the failed humor condition

	Variable	Mean	SD	1	2	3	4	5	6	7	8	9	10	11
Time 1	1. Gender	0.75	0.43	–										
	2. Trait PA	3.47	0.83	0.09	0.86									
	3. Trait NA	2.35	0.80	0.12	−0.47**	0.90								
	4. Trait guilt	2.05	0.92	0.05	−0.43**	0.71**	0.79							
	5. Affective PT	3.56	0.95	−0.01	0.43**	−0.23	−0.27*	0.89						
	6. Humor ability	3.44	1.00	−0.25	0.39**	−0.27*	−0.29*	0.38**	0.93					
Time 2	7. State guilt	2.22	1.18	−0.26*	−0.23	0.43**	0.52**	−0.30*	−0.14	0.86				
	8. New attempt	0.15	0.33	−0.09	0.18	−0.07	−0.35**	0.33**	0.32**	−0.30*	–			
	9. Laughed	0.13	0.34	−0.01	0.13	−0.15	−0.16	0.29*	0.19	−0.26*	0.27*	–		
	10. Changed subject	0.10	0.30	0.06	−0.17	0.14	0.01	−0.22	−0.05	0.21	−0.09	0.04	–	
*n* = 61	11. Humor ability	3.23	1.08	−0.28*	0.35**	−0.14	−0.21	0.31*	0.84**	−0.12	0.37**	0.20	−0.01	0.92

Men were coded as 0, women as 1

* *p* > 0.05; ** *p* < 0.01

**Table 2:** Descriptive statistics and zero-order correlations for the successful humor condition

	Variable	Mean	SD	1	2	3	4	5	6	7	8	9	10	11
Time 1	1. Gender	0.74	0.44	–										
	2. Trait PA	3.51	0.73	−0.09	0.86									
	3. Trait NA	2.40	0.72	−0.06	0.03	0.90								
	4. Trait guilt	2.32	0.96	−0.10	−0.20	0.71**	0.79							
	5. Affective PT	3.62	0.83	−0.20	−0.06	−0.11	−0.01	0.89						
	6. Humor ability	3.48	0.92	−0.05	0.35**	0.04	−0.06	−0.17	0.93					
Time 2	7. State guilt	1.16	0.37	−0.03	−0.21	0.11	0.20	0.01	−0.14	0.86				
	8. New attempt	0.19	0.40	0.13	−0.11	−0.12	−0.05	0.14	−0.08	0.06	–			
	9. Laughed	0.51	0.50	0.04	−0.12	−0.23	−0.18	−0.06	0.02	−0.07	0.19	–		
	10. Changed subject	0.03	0.18	0.11	0.04	0.14	0.05	−0.11	−0.03	−0.09	−0.09	0.08	–	
*n* = 66	11. Humor ability	3.16	0.89	−0.08	0.14	0.10	0.04	0.13	−0.08	−0.14	−0.14	0.03	−0.11	0.92

Men were coded as 0, women as 1

*p* > 0.05; ** *p* < 0.01

#### Goals and Type of Failed Versus Successful Humor: Recall Biases?

First, we investigated the goals and type of humor to assess whether any recall bias appeared when individuals recalled failed humor. To do this, we looked for systematic differences in the types and goals of humor associated with recalling a successful versus a failed humor attempt. We conducted a series of logistic regressions to investigate whether humor type had a significantly different role in influencing the type and goals of failed versus successful humor attempts. We used the Wald *χ* ^2^ statistic as an indication of the influence of condition because it tests the unique contribution of an independent variable to influence a focal binary outcome variable. First, describing a successful or failed humor attempt did not influence the rate at which participants recalled an instance of negative humor, Wald *χ* ^2^ = 0.43, *p* = 0.51. Specifically, about one-third of participants describing both failed and successful humor attempts used negative humor. Additionally, gender did not affect humor type, Wald *χ* ^2^ = 2.66, *p* = 0.10. Similarly, the goals of successful and failed humor did not differ as indicated by our logistic regression analyses. In both conditions, approximately 78 % of participants made a humor attempt to raise the target’s general positive affect, Wald *χ* ^2^ = 0.01, *p* = 0.97 and 16 % of participants made their attempt to strengthen their positive affective bond to the target, Wald *χ* ^2^ = 0.43, *p* = 0.51. Thus, because people do not seem to recall different types of humor episodes when asked to recount a failed versus a successful humor attempt, we can be more confident that the results reported below are related to the experience of failed humor rather than to the experience of recalling a particular type of humor (negative versus positive) or to a particular humor goal that is associated with failed humor (i.e., generating positive affect versus strengthening positive affective bonds).

In addition, we have explicit evidence from coding of the narratives that 94 % of the respondents intended to regulate the internal feeling states of the targets as well as their outward expression of laughter. Those individuals who did not explicitly state a goal of regulating the internal feeling state of the target tended to have the goal of regulating the target’s emotional expression (i.e., making the person laugh), but we cannot determine conclusively whether or not they had the goal of regulating the internal feeling state of the target.^3^

#### Manipulation Check: Documenting Responses to Failed Versus Successful Humor

Next, we sought to uncover and document baseline information about failed humor. We report affective responses related to feelings and self-esteem followed by behavioral responses and finally cognitive responses related to attributions. We controlled for gender and ethnicity in all of our analyses.

#### Responses to Failed Versus Successful Humor: Feelings, Self-Esteem, and Humor Self-Efficacy

Whereas the type and goal of failed versus successful humor did not differ, the outcomes of recalling a failed versus successful attempt were markedly distinct. For these analyses, the failed humor condition was coded as 1 and the successful humor condition was coded as 0. First, linear regression indicated that after recalling a failed attempt, agents were more likely to report experiencing negative affect, *β* = 0.39, *t* = 4.33, *p* < 0.01, *R* ^2^ = 0.22, and guilt, *β* = 0.51, *t* = 6.26, *p* < 0.01, *R* ^2^ = 0.31, than those recalling a successful attempt, and less likely to report positive affect, *β* = −0.74, *t* = 11.39, *p* < 0.01, *R* ^2^ = 0.57. Additionally, participants who had recalled a failed attempt were more likely to describe lower self-esteem, i.e., rating themselves as more stupid, undesirable, unlikeable, unattractive, incompetent, and foolish, *β* = 0.60, *t* = 7.50, *p* < 0.01, *R* ^2^ = 0.39, and were less likely to rate themselves highly on the opposing, positive self-esteem items, i.e., intelligent, likeable, wise, attractive, competent, desirable, than those who had recalled a successful attempt, *β* = −0.60, *t* = 7.19, *p* < 0.01, *R* ^2^ = 0.37.

#### Behavioral Responses to Failed Versus Successful Humor

Participants behavioral responses also differed based on the outcome of their humor attempt. A series of logistic regressions indicated that failed humor attempts were more likely to result in apologies, Wald *χ* ^2^ = 4.01, *p* = 0.04, changing the subject, Wald *χ* ^2^ = 4.50 *p* = 0.03, and doing nothing, Wald *χ* ^2^ = 7.16, *p* < 0.01, whereas they were less likely to result in the agent’s laughter, Wald *χ* ^2^ = 16.84, *p* < 0.01. Additionally, of importance, there were no differences in the numbers of new attempts made between conditions, Wald *χ* ^2^ = 0.01, *p* = 0.94; however, humor success and humor self-efficacy interacted to predict new humor attempts, Wald *χ* ^2^ = 7.01, *p* < 0.01. Specifically, those participants with high humor self-efficacy were more likely to make a subsequent attempt after failure, whereas no differences arose in the success condition.

#### Cognitive Attributions for Failed Versus Successful Humor

We also used logistic regression to document whether self-serving differences existed in people’s attributions for the causes of failed versus successful humor attempts. Whereas only 30 % of participants attributed failed humor to themselves, 51 % personally took credit for a successful attempt, Wald *χ* ^2^ = 5.08, *p* = 0.02. Conversely, a full 70 % of participants laid the blame for a failed attempt on the target, whereas only 33 % of participants gave the target credit for a successful attempt, Wald *χ* ^2^ = 14.60, *p* < 0.01. These patterns were consistent with a self-serving bias. There were no differences in attributions based on the specific method used (e.g., physical prank, teasing).

#### The Effect of Failed Humor on the Target of the Humor Attempt

In addition to coding the narratives for the behavior of the agents, we also went through them to investigate the recalled differences in the effects of failed versus successful humor had on the target of the humor attempt. The first, more obvious findings, indicated that failed humor attempts were associated with less target laughter, Wald *χ* ^2^ = 15.10 *p* < 0.01, and perceptions of being more bothersome, Wald *χ* ^2^ = 7.08 *p* < 0.01. Additionally, the failed humor condition was negatively associated with the target’s likely of building on the interaction or responding to the target at all, Wald *χ* ^2^ = 4.13, *p* = 0.04. This stresses the importance of successful interpersonal affect regulation in maintaining bonds with others.

### Inductive Results for Failed Humor

Next, we conducted a series of analyses to explore the main focus of our study: reactions to failed humor. We investigated how individual differences in gender, affective perspective taking, and trait guilt were related to the experience of failed humor and how these experiences differed from those of successful humor. Explicitly, we sought to understand how these individual differences mitigated or exacerbated responses to failed humor and how they were associated with (1) an agent’s willingness to persist in attempts to regulate the positive affect of others after failed humor, (2) an agent’s propensity to laugh following a humor attempt, (3) an agent’s feelings of state guilt after a humor attempt, and (4) an agent’s humor self-efficacy after a humor attempt. Again, we focused on an agent’s own laughter as the expected outcome of humor, guilt as a likely outcome offending a target during a failed humor attempt, and humor self-efficacy as a type of domain-specific confidence that is likely to decrease following a failed humor attempt. Participant’s willingness to make new humor attempts was investigated because it represents an explicit attempt to continue the interaction with a target after a failure of interpersonal affect regulation. To do this, we conducted regression analyses on the full sample. We investigated the relationship between individual differences measured during Phase 1 and psychological experiences and behavioral responses reported during Phase 2. Ethnicity and gender were included in all analyses.

We then included an interaction term between the individual difference variables/control variables and successful versus failed humor condition to determine the effect in the failed humor condition relative to the successful humor condition. In our analyses, significance on the interaction term reflects the difference in slope between the two conditions, whereas the significance level of the primary term reflects the effect of that primary term in the condition coded as zero (Aiken et al. 1991). The results of these analyses with humor condition coded, Success = 0, Failure = 1, are shown are shown in  3, 4 and 5 so that the significance of the coefficient on the primary term for each individual difference will reflect the significance in the successful condition (i.e., when success = 0) and the interaction term will reflect the change in the slope of the outcome regressed on the individual difference measure in the failure condition (i.e., failure = 1). Although not in the tables, we also reverse coded the humor condition coded, Success = 1, Failure = 0. Below, we report the significance of the primary term for each individual difference in both the failed condition and the successful conditions.

**Table 3:** Association of participant’s state guilt with trait guilt, affective perspective taking, and gender

Time 1 measures	Time 2 State guilt			
Affective perspective taking	−0.10	0.01	−0.09	−0.07
Trait guilt	0.31**	0.29**	0.31**	0.06
Gender (women = 1, men = 0)	−0.19*	−0.18*	−0.01	−0.19**
Success/Fail (Success = 0, Fail = 1)	0.55**	0.90**	0.85**	−0.01
Asian	0.05	0.03	0.03	0.01
White	0.13	0.08	0.09	0.06
Success/Fail × affective PT		−0.38		
Success/Fail × gender			−0.39**	
Success/Fail × trait guilt				0.63**
*R* ^2^	0.41	0.44	0.42	0.46

Numbers for state guilt represent *β* values as these variables were continuous. *n* = 127 for all full sample analyses

* *p* < 0.05; ** *p* < 0.01. *n* = 127

**Table 4:** Association of participant’s humor efficacy with trait guilt, affective perspective taking, and gender

Time 1 measures	Time 2 humor efficacy			
Affective perspective taking	0.07	−0.16	0.07	0.07
Trait guilt	−0.18*	−0.15	−0.16	−0.15
Gender (women = 1, men = 0)	−0.22*	−0.23**	−0.14	−0.21
Success/Fail (Success = 0, Fail = 1)	0.03	−0.78*	0.15	0.08
Asian	−0.26*	−0.23	−0.24	−0.24
White	−0.22	−0.18	−0.20	−0.20
Success/Fail × affective PT		0.85*		
Success/Fail × gender			−0.17	
Success/Fail × trait guilt				−0.06
*R* ^2^	0.13	0.17	0.13	0.13

Numbers for humor efficacy represent *β* values as these variables were continuous. *n* = 127 for all full sample analyses

* *p* < 0.05; ** *p* < 0.01. *n* = 127

**Table 5:** Association of participant’s new attempts with state guilt, humor efficacy, and gender

Time 1 measures	Time 2 new attempts		
Affective PT	3.92*	3.76*	1.99
Trait guilt	1.03	2.66	2.93^+^
Gender (women = 1, men = 0)	0.40	0.40	0.25
Success/Fail (Success = 0, Fail = 1)	0.01	2.28	6.45**
Asian	0.01	0.04	0.01
White	0.08	0.02	0.08
State guilt		2.16	
Humor efficacy			1.08
Success/Fail × state guilt		3.51^+^	
Success/Fail × humor efficacy			6.28**
*R* ^2^	0.07	0.19	0.23

Numbers for new attempts represent Wald *χ* ^2^ values and these variables were binary. The *R* ^2^ values for the binary variables are Nagelkerke *R* ^2^

^+^ *p* < 0.10; * *p* < 0.05; ** *p* < 0.01. *n* = 127

#### State Guilt

First, there was a significant interaction of trait guilt and humor condition on state guilt, *β*_Failure × Trait_ = 0.63, *t* = 3.33, *p* < 0.01, such that slope of state guilt on trait guilt was 0.63 greater in the failed condition. Trait guilt was not significantly related to state guilt in the successful condition as shown by the coefficient on the primary term when (Success = 0), *β*_Trait Guilt_ = 0.06, *t* = 0.62, *n.s.* (see Table 3). We also reverse coded the Success/Fail variable such that Failure = 0. We found that trait guilt was significantly related to state guilt in the failed condition as shown by the significant coefficient on the primary term when Failure = 0, *β*_Trait Guilt_ = 0.55, *t* = 7.15, *p* < 0.01.

Additionally, there was a significant interaction effect of gender and humor condition on state guilt, *β*_Failure × Female_ = −0.39, *t* = 2.30, *p* = 0.02, such that the slope of trait guilt on state guilt was 0.39 less for women in the failed condition. Gender was not significantly related to state guilt in the successful condition as shown by the coefficient on the primary term when Success = 0, *β*_Female_ = −0.01, *t* = −0.09, *n.s.* (see Table 3). In addition, we found that the intercept for being female was significantly and negatively related to state guilt in the failed condition as shown by the coefficient on the primary term when (humor condition coded as failure = 0) *β*_female_ = −0.35, *t* = 3.42, *p* < 0.01.

#### Humor Self-Efficacy at Time 2

Humor condition and affective perspective taking significantly interacted to influence humor self-efficacy after reporting the attempt, *β*_Failed × Affective PT_ = 0.85, *t* = 2.25, *p* = 0.03 such that affective perspective taking was associated with humor efficacy in the failed, *β*_Affective PT_ = 0.24, *t* = 2.01, *p* < 0.05, but not in the success condition, *β* = −0.16, *t* = 1.18*, n.s.* (Table 4). Trait guilt did not interact with humor condition and was not related to humor efficacy after controlling for the interaction between humor condition and affective perspective taking. Being female appeared to be negatively related to humor efficacy across successful and failed conditions. However, further analysis revealed that the relationship between gender and humor efficacy was more complex.

**Fig. 1:**
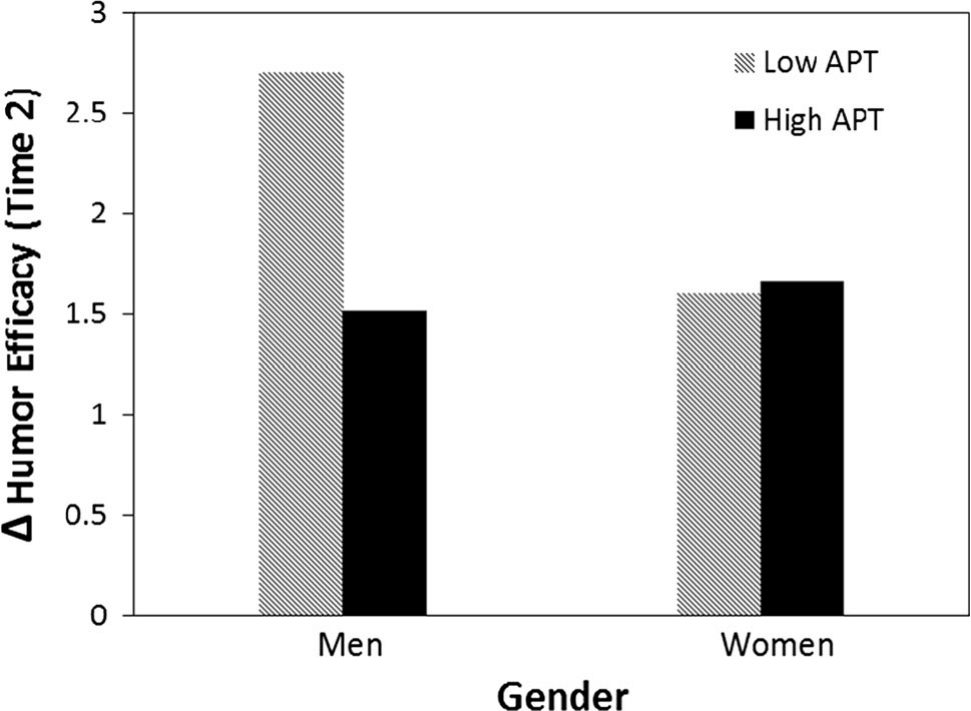
The moderation of affective perspective taking on the relationship between gender and humor self-efficacy following a failed humor attempt

**Fig. 2:**
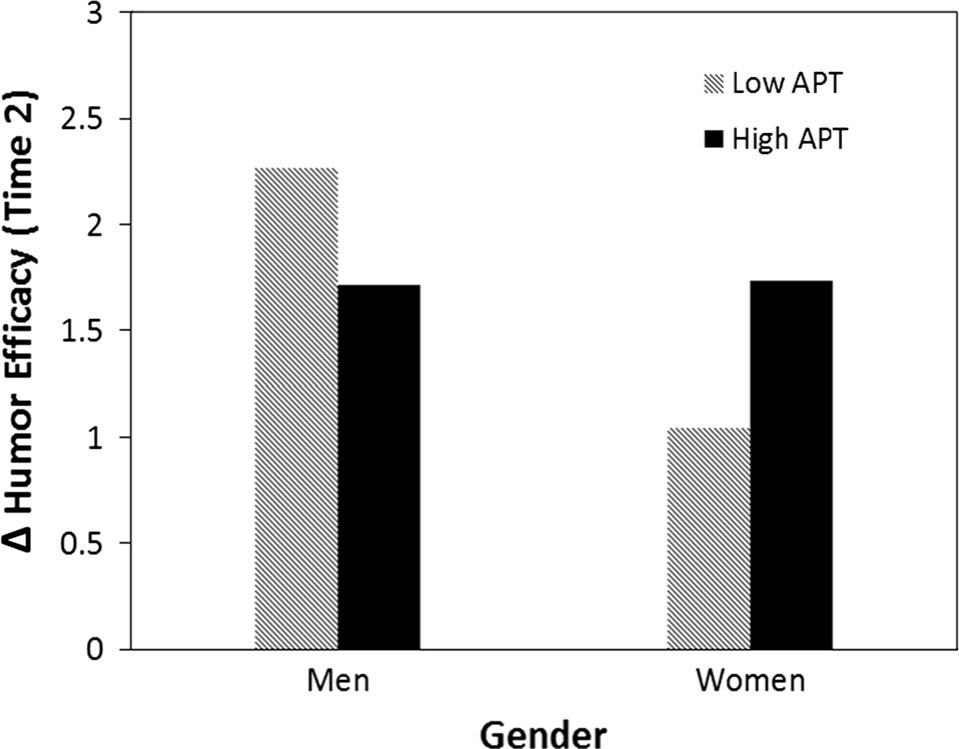
The moderation of affective perspective taking on the relationship between gender and humor self-efficacy following a successful humor attempt

To address this, we conducted a second set of regressions that indicated that gender and affective perspective taking interacted to influence humor efficacy, *β*_women × Affective PT_ = 0.70, *t* = 3.05, *p* < 0.01 (Figs. 1 and 2). The interaction between humor condition and affective perspective taking remained marginally significant in these analyses, but the interaction between gender and humor condition was not significant nor was there a significant 3-way interaction among humor condition, gender, and affective perspective taking.

Interestingly, affective perspective taking was only significantly related to men’s humor efficacy in the failed condition and women’s humor efficacy in the success condition. For men, high-trait affective perspective taking was associated with decreases in perceptions of their own humor ability after failure relative to low-trait perspective taking (Fig. 1). For women, high-trait affective perspective taking was associated with increases in humor self-efficacy after success relative to low-trait perspective taking (Fig. 2). Further, in both the failed and successful humor conditions, high trait perspective taking brought men and women’s perceptions of their humor ability into alignment, suggesting the possibility that high perspective taking allowed men to make more accurate downward adjustments to their humor efficacy after failure, and women to make more accurate upward adjustments to their humor efficacy after success.

#### Agent’s Own Laughter

A similar analysis was conducted to test the influence of trait guilt and affective perspective taking on agents’ tendency to laugh in spite of a failed attempt. In this instance, the analysis indicated that neither affective perspective taking nor trait guilt were associated with laughter and neither interacted with condition to influence an agent’s own laughter, Wald *χ* ^2^ = 0.04, *n.s.* and Wald *χ* ^2^ = 2.29, *n.s.*, respectively.^4^

#### Willingness to Make a New Humor Attempt

In order to investigate the relative influence of trait guilt and affective perspective taking on participant’s willingness to make a new humor attempt, this willingness was logistically regressed on both variables.

Results of the analysis indicated that affective perspective taking increased participant’s tendency to make a new attempt after both success and failure, Wald *χ* ^2^ = 3.92, *p* = 0.04 (see Table 5). The interaction between affective perspective taking and success versus failure condition was not significant Wald *χ* ^2^ = 0.04, *n.s.* nor was the interaction between trait guilt and success versus failure condition Wald *χ* ^2^ = 2.29, *n.s.*

However, the willingness to make new attempts was related to two of the outcome variables in the failed condition: humor efficacy at Time 2 and state guilt (Table 5). There was a significant interaction between humor efficacy at Time 2 and being in the success or failure condition Wald *χ* ^2^ = 6.28, *p* = 0.01. Humor efficacy at Time 2 was significantly related to new attempts in the failure condition Wald *χ* ^2^ = 5.87, *p* = 0.02, but not in the success condition Wald *χ* ^2^ = 1.08, *n.s.* There was also a marginally significant negative interaction between state guilt at Time 2 (after experiencing the success or failure condition) and being in the success or failure condition Wald *χ* ^2^ = 3.51, *p* = 0.06.

### Supplemental Analyses

#### SEM Analyses of Individual Differences After Failed Humor

**Fig. 3:**
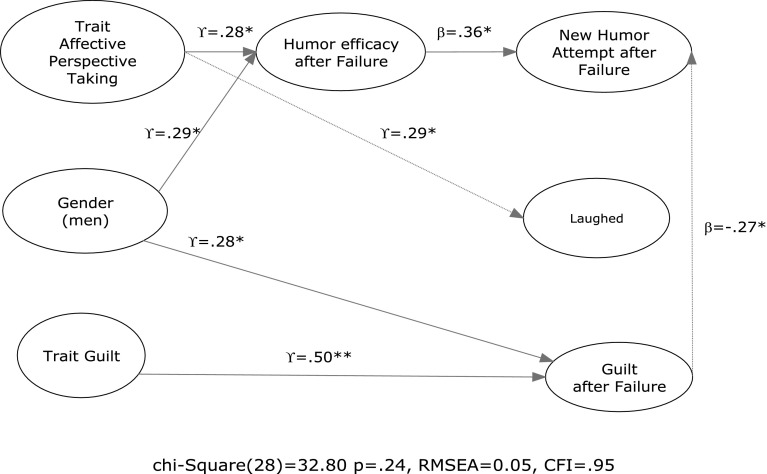
SEM analysis of failed humor (*Model controls for trait positive affect, trait negative affect, and behavioral response of “changing the subject”)

To further investigate the findings, we conducted a path analysis on the variables above (i.e., a fully aggregated structural equation model) using the data from the failed humor condition (Fig. 3). SEM allowed us to examine multiple dependent variables simultaneously and look for mediation effects that were suggested by our previous analyses (Kline 2005). Using Lisrel 8.8 software (Jöreskog and Sorbom 1997), we found that the SEM model fit well overall (χ(28) = 32.80, *p* = 0.24, RMSEA = 0.05, CFI = 0.95) and that the parameter estimates were consistent with the regression results presented above. We found that after controlling for trait positive affect (T1), trait negative affect (T1), and the behavioral response of changing the subject (i.e., no paths estimated), gender was positively related to humor self-efficacy (*γ* = 0.29, *p* < 0.05) and state guilt after failure (*γ* = 0.28, *p* < 0.05); trait guilt (T1) was positively related to state guilt (T2) (*γ* = 0.50, *p* < 0.01); state guilt (T2) was negatively and directly associated with making a new attempt (*γ* = −0.27, *p* < 0.05). Additionally, humor efficacy was positively and directly related to making a new attempt (*β* = 0.36, *p* < 0.05), whereas the influence of affective perspective taking on new attempts seemed to be indirect and mediated by humor efficacy (i.e., a direct effect on humor efficacy, *γ* = 0.28, *p* < 0.05), which in turn was related to new attempts. Affective perspective taking was also positively related to laughter after a failed attempt (*γ* = 0.29, *p* < 0.05).

**Fig. 4:**
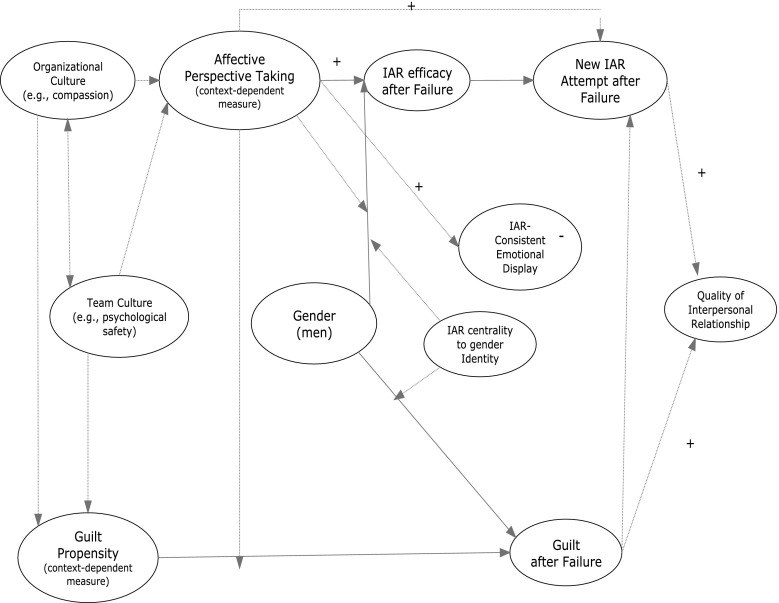
Inductive model of failed interpersonal affect regulation (*Model assumes controls for trait positive affect and trait negative affect)

Because affective perspective taking (T1) was associated with new attempts in our regression analyses, we also tested a model freeing the path from affective perspective taking (T1) to new attempts (T2). A sequential Chi square difference test indicated that the revised model did not fit significantly better than our original model (Δχ(1) = 2.11, *p* = 0.15), so we retained the more parsimonious model. Thus, although we cannot test our model on the same data used to develop it, the results presented here are suggestive of a more comprehensive set of simultaneous relationships summarized in Fig. 4.

It is important to note that our sample size was too small to conduct a reliable robustness test using the full data set and multiple group analyses. However, the suggestive multiple group analyses that we conducted confirmed the results presented here. The estimated paths in Fig. 3 were significant in the failed condition and non-significant in the success condition.

## Discussion

In today’s society, telling a “bad joke,” that is, a joke that is simplistic, silly, and likely to fail, has become humorous in and of itself. Numerous websites advertise lists of “Bad Jokes,” “Bad Jokes of the Day,” and “Really Bad Jokes” (https:/www.google.com, search term “bad jokes”).^5^ However, our study suggests that failed humor is no laughing matter. Failed humor, and more broadly the failed interpersonal regulation of positive affect, may not only lead to forgoing the potential benefits of positive affect such as helpful, prosocial behavior, enhanced decision making, and creativity (Fredrickson 2001; Isen 1987, 2008), but also may result in negative affect, decreased self-esteem, and the unwillingness to persist in affect regulation efforts. In other words, our results suggest the viewing affect regulation as a communicative process (that can either succeed or fail) has important implications for the study of humor in organizations.

Despite the commonality and importance of failed efforts at interpersonal affect regulation, inadequate research attention has been paid to this phenomenon. To address this oversight, we conducted an inductive study using humor as a lens to investigate how making failed versus successful attempts at interpersonal affect regulation was associated not only with agents’ feelings, cognitions, and behaviors, but also with how individual differences mitigated or conversely, exacerbated reactions to failed humor attempts.

Specifically, with our descriptive statistics and manipulation check, we both documented individuals’ responses to recalling failed humor and uncovered a noteworthy pattern of results. First, we investigated and documented whether recalling a failed humor attempt was a negative experience for the agent or whether humor was experienced as a “frivolous” method of interpersonal affect regulation such that individuals would take success or failure in stride. We found that recalling a failed humor attempt indeed was associated with decreased positive affect and self-esteem and increased guilt. Although we cannot disentangle affective responses to recalling failed humor from the general recall of failure, respondents’ reported behaviors at the time of the humor event that provide corroborating evidence of negative affective reactions. Agent’s reported a tendency toward avoidance behaviors associated with negative affect (e.g., doing nothing or changing the subject), which suggests it is likely that individuals experienced negative affect at the time of their failed interpersonal affect regulation (Chen and Bargh 1999; Markman and Brendl 2005).

Our findings suggest that making humor attempts can increase the positive affect in a relational interaction but can also risk reducing it. When interpersonal affect regulation is successful, our data suggest that the agent as well as the target, who typically laughed and was more willing to build on the interaction, experienced increased positive affect. Although beyond the scope of our study, this increase in positive affect may have important implications for agents. Research on positive affect consistently finds that positive affect can increase helpful, prosocial behavior, enhance decision making, and boost creativity (Isen 1987, 2008). Similarly, research on the successful regulation of positive affect suggests that agents can experience improved well-being and stronger relationships (Niven et al. 2012a, b). For these reasons, viewing interpersonal affect regulation as a process that can succeed or fail in increasing positive affect has implications for a variety of personal, interpersonal, and work-related outcomes.

### Implications of Our Inductively Derived Model

First, our inductively derived model suggests that affective perspective taking, trait guilt, interpersonal affect regulation self-efficacy, and gender, each play an important role in individuals’ experience of failed interpersonal affect regulation. In the specific case of humor, affective perspective taking and humor self-efficacy were associated with a higher likelihood of making a new humor attempt, whereas state guilt after failure was associated with a lower likelihood.

Future research could extend this work by investigating the mechanisms associated with each individual difference. For instance, because affective perspective taking is related to greater valuing of the well-being of others (Batson et al. 1995), it may be that individuals high in affective perspective taking were better able to remain focused on the target’s need for positive affect and their initial desire to help the target. In fact, in our sample, the correlation between affective perspective taking and new attempts was stronger for individuals with the goal of increasing the target’s general positive affect. Moreover, affective perspective taking was associated with a higher likelihood of making a new humor attempt after a successful as well as a failed humor attempt, suggesting that the benefits of affective perspective taking may extend to a wide variety of situations involving interpersonal affect regulation.

We also uncovered an efficacy-based process related to interpersonal affect regulation. Humor self-efficacy had a positive relationship to new humor attempts, but only after failure, suggesting that people’s self-efficacy related to humor and other specific domains of interpersonal affect regulation may be a critical aspect of their experiences of failed interpersonal affect regulation and their willingness to persist. This finding corroborates past research suggesting that domain-specific efficacy increases people’s tendency to persist in the face of failure (Bandura 1997). Importantly, the fact that humor self-efficacy may mediate the impact of affective perspective taking on new attempts after failure suggests that it is not only emotional intelligence (i.e., the emotion management dimension, Salovey and Mayer 1990), but also efficacy with respect to one’s emotional intelligence that may be critical for how people experience successful versus failed affect regulation attempts and how these processes unfold over time.

Our findings with respect to guilt were counterintuitive. Although guilt often prompts relationship repair (Baumeister et al. 1994), we found that people who felt guilty after failure (i.e., were high on state guilt) were slightly less likely to try a new attempt. This finding suggests that future research needs to examine how ignoring a faux pas might be a strategy for relationship repair that is as important to understand as the use of apologies and accounts. Perhaps such behavior allows the incident to “blow over” such that both the target and the agent can save face. The agent does not dwell on having made a failed attempt, and the target is allowed to avoid feeling inadequate for not understanding or responding as expected to the interpersonal affect regulation attempt.

Finally, we did find direct effects of gender on humor self-efficacy and state guilt. In terms of guilt, we believe that gender is likely to have an important role in interpersonal affect regulation more broadly but that the specific effects of gender may differ across different types of regulation. For instance, it is unclear whether men will always feel more guilt than women after failed interpersonal affect regulation attempts because relative to women, men are likely to rate as important both their own humor ability and other’s receptivity to their humor (Bressler et al. 2006). Thus, because humor is likely to be more central to the identity of men than women (Bressler et al. 2006), failing at humor may be associated with more guilt in men than women. However, affect regulation processes that are more central to the identity of women may have the inverse effect and, for example, be associated with greater guilt in women than men.

Future research could examine whether failing at interpersonal affect regulation strategies linked to empathy, for example, has a more negative effect on women’s emotional responses and a less negative effect on their affect regulation efficacy than it does on men’s. Empathy-related skills such as listening to another’s problems may be more important to women because empathic concern is associated with the stereotype of women and also socialized in young girls (Cross and Madson 1997; Eagly and Wood 1999). Moreover, if the impact of sex differences on the experience of failed interpersonal affect regulation is in fact moderated by the importance of the particular domain of affect regulation, our finding could open up a broader investigation of identity concerns within the context of interpersonal affect regulation.

### Moderation: Affective Perspective Taking, Gender, and Humor Efficacy

Because of the benefits of maintaining positive affect, we were also interested in factors that might buffer individuals against the negative effect of failed interpersonal affect regulation. Our humor findings suggest that affective perspective taking may operate in this way. Moreover, the interaction between gender and affective perspective taking provides nuance to our general finding that women seem to show significantly less humor efficacy after both failed and successful humor attempts.

In the case of failed humor, perspective taking did not influence women’s humor self-efficacy after a failed attempt. However, low perspective taking buffered men from the decreased humor self-efficacy associated with their failed attempts, whereas high perspective taking exacerbated the negative impact associated such failure (Fig. 1). For high perspective takers, both men and women reported similar levels of humor efficacy associated with a failed attempt. Our findings suggest that when perspective taking is low, overconfidence may buffer men from the influence of failed humor on their beliefs in their humor self-efficacy. However, when perspective taking is high, both men and women perceive their humor self-efficacy similarly after failure.

Under the condition of humor success, we found gender effects for women but not men. Affective perspective taking did not influence men’s humor efficacy after success. However, for women, high perspective taking was related to significantly greater humor efficacy after success. In the successful humor condition, high perspective taking women reported similar levels of humor efficacy to men.

In other words, perspective taking seems to allow men and women to more appropriately adjust their efficacy beliefs. Appropriate adjustments upward should increase individuals’ willingness to persist in interpersonal affect regulation efforts, whereas appropriate adjustments downward may provide a better chance to succeed in future interactions, instead of making similar errors or miscalculations. Moreover, this finding allows for the possibility that the opposite pattern of results could be found for other methods of interpersonal affect regulation that may be more important to the identity of women and for which women are more confident than men.

### Implications for Practice

Humor is a ubiquitous and potentially beneficial part of organizational life. Katherine Hudson, former CEO of the Brady Corporation, contends that humor can “foster *esprit de corps*…spark innovation…increase the likelihood that unpleasant tasks will be accomplished… [and] relieve stress” (Hudson 2001 cited in Romero and Cruthirds 2006). Her comments are consistent with the well-researched benefits of humor and of positive affect, more generally, for decision making, creativity, and prosocial behavior (Fredrickson 2001; Isen 1987, 2008).

However, when managers tout the benefits of humor, they rarely consider the personal and interpersonal costs of failed humor attempts. Our study suggests that failed humor and the importance of employees’ willingness to persist and try again should be placed on the managerial radar. Specifically, because the successful interpersonal regulation of positive affect even after a failed attempt increases positive affect, persistence in such regulation attempts has implications for helping behavior, decision making and creativity in interdependent work relationships, project teams, and organizations as a whole.

Research on relationship-specific measures of perspective taking has further implications for organizations. This research suggests that relationship-specific and context-specific measures of perspective taking have a consistent but stronger effect on outcomes than do measures of dispositional measures of perspective taking (Davis 1996; Galinsky et al. 2008). Based on this research, we believe that it is not only people’s trait guilt and perspective taking that are likely to matter but also their relationship-specific and context-specific propensity to feel guilt and engage in perspective taking in their work versus home environments or with team members versus managers, for example. If, as we believe, context is important and context-specific measures of guilt and perspective taking will have similar effects to those we have shown here, then our findings suggest that managers can take either a group-level or an individual-level approach to facilitating the successful use of positive humor and interpersonal regulation of positive affect in the workplace.

At the group level, norms that decrease feeling of guilt over failure and increase compassion may increase individuals’ willingness to persist after a failed attempt at interpersonal affect regulation by influencing guilt and affective perspective taking, respectively. For example, team cultures that emphasize psychological safety (Edmondson 1999) and organizational cultures that foster love (Barsade and O’Neill 2014), forgiveness (Fehr and Gelfand 2012), and compassion (Lilius et al. 2011) may increase the degree to which individuals engage in context-specific and relationship-specific affective perspective taking, while at the same time reducing individuals’ tendency to associate guilt and blame with failure in the organizational context.

At the individual level, our model suggests that self-efficacy as it pertains to interpersonal affect regulation strategies may be of critical importance. In organizations, managers may use skill-based training to increase the degree to which individuals gain self-efficacy with respect to their use of a variety of effective strategies for managing their emotions and those of others (e.g., affective perspective taking, Williams 2011; reappraisal influence, Little et al. 2013; and humor, Niven et al. 2011). This may not only increase individuals’ willingness to persist in the regulation of other people’s positive affect, but also the ability to move seamlessly among different strategies rather than resorting to “doing nothing” as did many of our respondents.

## Limitations

Despite its inherent strengths, this study has several limitations. Autobiographical narratives are a useful tool for studying interpersonal phenomena such as *failed* humor that are difficult to recreate within the laboratory (Francis et al. 1999; Leary et al. 1998). Indeed, because “humor is a specific and easily recognizable form of interaction,” it is particularly amenable to recall-based methods such as autobiographical narratives (Francis et al. 1999, p. 156). Although the method we used in this research was appropriate for investigating how individual differences influence people’s subjective experience and behavioral responses to successful and failed interpersonal affect regulation, the inherent limitations of autobiographical narratives must be considered.

First, it is not possible to know the heuristics people used to recall humor episodes and whether these heuristics differed for successful and failed humor episodes. Although using autobiographical narratives has strengths, one drawback is that this methodology leaves open the possibility of recall biases. Although most respondents described events occurred in the past week, we gave them a window of up to 6 months to choose an event. Memories of the event may have changed over this time period. Despite this limitation, several steps were taken to investigate potential sources of bias in our study. First, the study was conducted in two phases to minimize the influence of Time 1 answers on the Time 2-dependent measures. Additionally, the type of humor used and the motivations for using humor were coded by independent coders blind to the purpose of the study and then compared across the failed and successful humor conditions to look for possible recall biases. As stated, no differences were found between these variables in our two conditions, supporting the premise that the participants’ ratings on the survey scales were likely related to recalling failed versus successful humor rather than biased recall of a particular a type of humor event (positive versus negative) or a particular motive associated with engaging in the humor attempt.

Second, self-serving biases are always present to some degree in autobiographical narratives. However, this does not necessarily mean that the general pattern of results does not apply (Leary et al. 1998). The fact that the narratives were related in a systematic way to individual differences measured a week before the narratives were written and to content analysis of the narratives by independent coders blind to the purpose of the study provides converging evidence and promotes confidence in the reliability of our participants’ self-reports. Thus, despite the limitations of the use of narratives, we believe that this study offers an important step toward understanding failed interpersonal affect regulation.

The fact that humor can be used to regulate affect in two different ways is both strength and a limitation of this study. Humor may be used in a response focused manner to change target’s outward emotional expression, i.e., “to get people to laugh” or to try to change target’s inner feeling state, i.e., to make them feel better. In a study of humor, these goals are intertwined. Agents who want to change targets’ internal states also want them to show a visible sign of feeling better, i.e., to laugh and smile. Conversely, agent’s who only want targets to laugh are also likely to change the internal state of those targets because the muscle movement associated with laughter and genuine smiling improve affect (Buck 1980). Although most of our respondents (94 percent) explicitly reported the goal of trying to change the target’s inner feeling state, for the other respondents, it is unclear whether they were operating with this goal in mind. It is possible that they used humor solely to “make people laugh.” This would regulate affect but also might generate more self-relevance for the agent with respect to success or failure. However, we found no empirical evidence that having the explicit goal of changing the target’s affect versus not explicitly having this goal was significantly correlated with agents’ attributions, feelings, self-esteem, or behavior after recalling the incident. Further, the failure condition did not seem to make respondents less likely to recall and report the other-oriented goal of changing the target’s positive affect. We would expect this difference in goals if the failure condition narrowed respondents’ focus to their own humor ability, appearing funny and “getting a laugh.” However, respondents in the failed humor condition were no less likely to explicitly report the goal of changing the target’s affect than respondents in the successful humor condition.

Finally, our study is limited to examining one type of interpersonal affect regulation, humor. Although humor is ideal for the study of failed interpersonal affect regulation because agents receive immediate and clear feedback about the success or failure of their attempt, it also may limit the generalizability of the study to other methods of interpersonal affect regulation. Of particular concern are methods of interpersonal affect regulation that are not likely to be as central to an agent’s self-concept as humor is likely to be. For example, reappraisal is a strategy that can be used to improve or worsen affect and may not be highly self-relevant. However, a range of affect-improving strategies such as complimenting and listening (Niven et al. 2009) are likely to tap into characteristics such as niceness, helpfulness, and empathy that are often central to individuals’ identities, especially individuals with relational self-concepts (Cross et al. 2000; Cross and Madson 1997). Thus, our findings may be most applicable to affect-improving strategies that are self-relevant. Future research should investigate failing at affect-worsening strategies such as aggression that are likely to be antithetical to maintaining a relational self-concept but consistent with other positive self-representations (e.g., gender stereotypes for men, especially those working in male-dominated industries).

## Conclusion

Despite emotions being a ubiquitous part of social life, until recently, the study of interpersonal affect regulation has lacked a common framework (Niven et al. 2009). One consequence of this disparity has been that *failed* interpersonal affect regulation and its cognitive, affective, and behavioral consequences has been largely ignored. However, our work shows that the investigation of interpersonal affect regulation as a process that can succeed or fail is vital to understanding how it affects people in social environments. Failure to regulate another person’s positive affect has negative consequences for the agent and potentially for his or her relationship with the target, while successful attempts not only have positive consequences for the agent but are likely to generate additional benefits associated with increased positive affect such as improved decision making. Because of this, it is hoped that the current study is viewed as a first step in promoting a new perspective on interpersonal affect regulation—one that allows for the possibility of failure. Bringing the possibility of failure to center stage allows researchers to investigate and managers to address the psychological processes that mitigate and, conversely, exacerbate the negative impact of these failures as well as factors that motivate persistence in the regulation of interpersonal positive affect and processes that enhance the effectiveness of interpersonal affect regulation.

## Footnotes

1 Actions such as teasing or sarcasm are often intended to be humorous to a target individual or to a target group observing a dyadic interaction. However, teasing and sarcasm may be used for the purposes other than humor such as criticism or aggression (Keltner et al. 2001). Our study is restricted to the subset of such behaviors that are intended to influence positive affect.

2 Men and women were randomly assigned to the failed versus the successful humor conditions (*N*_failed_ was 74 % female and *N*_success_ was 75 % female).

3 We did, however, investigate mean differences in responses to humor incidents between individuals who did and did not explicitly report this additional goal. There were no mean differences found between the two groups in the successful humor condition. In the failed humor condition, individuals with explicit goals of interpersonal affect regulation reported higher mean levels of positive affect after failure and had higher means for describing new and repeated humor attempts in their narratives. However, these mean differences did not translate into a significant correlation between having explicit affect regulation goals and any other variables including positive affectivity and making new or repeated humor attempts after reporting about failed humor.

4 When we controlled for state guilt in our regresssion analyses, as happened automatically in the SEM analyses in the following section, affective perspective taking had a significant positive interaction with condition such that it had stronger relationship to an actor’s own laughter in the failed condition (Success/Fail × Affective PT, *Wald* *χ* ^2^ = 3.60*, *p* < 0.05).

5 A sample “bad joke” follows (http://www.rinkworks.com/jokes/, joke #882): Actor: “What did the fisherman say to the card magician? Target: “What?” Actor: “Pick a cod, any cod!”
